# Staged transarterial and transvenous embolization of a high-grade ruptured tentorial dural arteriovenous fistula

**DOI:** 10.3171/2025.7.FOCVID2593

**Published:** 2025-10-01

**Authors:** William M. Burns, Santiago Mendoza-Ayus, Allison Young, Pablo V. Barrera, Gurkirat S. Kohli, Prasanth Romiyo, Rohin Singh, Tarun Bhalla, Matthew T. Bender, Thomas K. Mattingly, Vincent N. Nguyen

**Affiliations:** 1 Department of Neurosurgery, University of Rochester Medical Center, Rochester; and; 2 Rochester Institute of Technology, Rochester, New York

**Keywords:** tentorial dural arteriovenous fistula, *N*-butyl cyanoacrylate, staged embolization, transarterial embolization, transvenous embolization

## Abstract

Tentorial dural arteriovenous fistulas (TDAVFs) are rare but high-risk lesions due to frequent deep venous drainage. A 77-year-old woman presented with a ruptured, high-grade (Borden III, Cognard IV, Zipfel 3S) TDAVF at the pontomesencephalic junction. Angiography revealed feeders from the left middle meningeal artery, occipital artery, and meningohypophyseal trunk with tortuous venous drainage into a partially thrombosed varix. Initial transarterial *N*-butyl cyanoacrylate (*N*-BCA) embolization was incomplete. Definitive cure was achieved with transvenous coiling. Postoperatively, the patient was neurologically stable, and follow-up angiography revealed no further arteriovenous shunting, demonstrating how staged embolization can lead to curing TDAVFs with complex angioarchitecture.

The video can be found here: https://stream.cadmore.media/r10.3171/2025.7.FOCVID2593

## Transcript

This is a case of a staged transarterial and transvenous endovascular embolization for treatment of a high-grade ruptured tentorial dural arteriovenous fistula.

### 0:31 Clinical Presentation.

The patient is a 77-year-old female who presented with severe headaches and vomiting. Her history is significant for COPD on home oxygen, as well as obesity with a gastric sleeve operation and other comorbidities. On initial exam she was mildly lethargic with some upper extremity ataxia and was subsequently intubated for inability to protect her airway.

### 0:58 Neuroimaging Findings and Control Angiography.

Her noncontrast CT scan demonstrates bilateral superior cerebellar intracerebral hemorrhage as well as quadrigeminal cistern subarachnoid hemorrhage. Her T2 axial MRI demonstrates a venous varix at the level of the pontomesencephalic junction. Her time-of-flight MRA demonstrates tentorial arterial feeders to a deep venous varix with deep venous drainage. Here you can see our initial run at the first stage. This is the left external carotid artery injection, demonstrating the middle meningeal artery feeders, as well as the extremely tortuous occipital artery feeder with direct fistulization into a cortical vein with symptomatic presentation from intracranial hemorrhage, making it a Zipfel 3S dural arteriovenous fistula. There is no supply of significance from either vertebral artery or the right internal or external carotid circulation.

### 2:04 Rationale for the Procedure.

The rationale for the procedure was that this was a ruptured, unsecured, high-grade dural arteriovenous fistula. Given her frailty, she initially was a DNR, but after discussion with family the order was reversed for the procedure. The risks and benefits were thoroughly discussed with family, including hemorrhage, stroke, death, and benefits for protection from further fistula rupture. We considered alternatives including microsurgical treatment through an extended retrosigmoid approach. However, we favored an endovascular approach first, given her age, medical comorbidities, and current condition.

### 2:41 Stage 1 Transarterial Embolization.

For the arterial stage, we utilized a 6-French femoral sheath and the catheter setup as described, utilizing NBCA Trufill glue liquid embolic agent. Another option for use of liquid embolic would have been Onyx to achieve penetration into the fistula. Here’s a microcatheter injection during our first stage transarterial approach of the middle meningeal artery petrous branch. We obtained distal access and, after NBCA embolization, there was persistent fistulous filling. Here you can see the glue cast on the nonsubtracted run. Further transarterial microcatheterization and embolization was deemed unsafe and unfeasible. The occipital artery was another potential target, but given its significant tortuosity, this was not deemed to be favorable.

### 3:34 Stage 2 Transvenous Embolization.

Several days later, a second-stage transvenous approach was performed, obtaining access to the straight sinus using a SOFIA over XT-27 over Aristotle 24, subsequently exchanging for a Headway Duo 156 over Synchro-soft, and going "around the world" through the unruptured venous varix to access the mouth of the fistula for transvenous coiling.

Here’s a left internal carotid artery injection at the second stage, demonstrating a small feeder from the meningohypophyseal trunk. Here’s a control run from the left external carotid, demonstrating continued filling through the occipital and middle meningeal arteries. A 3D rotational angiogram was performed from the left external carotid artery, here shown with some reflux into the internal carotid artery. Cone beam CT angiography using the DynaCT software demonstrates the complex anatomy of this ruptured dural fistula with drainage through the basal vein of Rosenthal and lateral mesencephalic vein. There’s an unruptured venous varix as well as a partially thrombosed portion that was thought to be the rupture point. The fistulous pouch is seen here adjacent to the petrous bone. Here are the transosseous occipital artery feeders noted. Here is a 3D artistic reconstruction of this fistula demonstrating the partially thrombosed varix, the fistulous point, as well as the large unruptured varix and subsequent deep venous drainage. These are treatment angles here for transvenous coiling. A careful study of these preoperative reconstructions is critical to allow us to plan for coil embolization of the exact fistulous point. Here is a control left external carotid artery injection after transvenous access has been obtained. Here we are beginning navigation through the deep venous system with significant tortuosity and loading forces noted on the SOFIA 125 Intermediate. At double speed here, you can see microwire manipulation through the significantly stenotic and tortuous deep venous drainage with the goal of obtaining access to the unruptured venous varix. The Synchro-14 is then advanced in an "around the world" fashion through the deep venous varix to obtain additional purchase and support to allow for navigation of the Headway Duo 156 to the junction of the unruptured venous varix and fistula. The microwire was advanced into the mouth of the fistula; however, despite multiple attempts, the microcatheter would not track over the microwire. Here, using digital magnification, we advance the microcatheter past the fistulous point into the unruptured venous varix to obtain additional distal purchase past the mouth of the fistula. The Headway Duo microcatheter is subsequently reduced slowly to obtain access directly into the mouth of the fistula. We then deploy a Target XL 3 mm × 6 cm coil directly into the mouth of the fistula and partially into the ruptured portion of the varix. A second coil 2 mm × 6 cm Target XL is then deployed with progressive stasis now demonstrated in the venous varices. Finally, a 5 mm × 10 cm Target XL is deployed partially into the fistula and mostly into the unruptured venous varix, however, with good deployment overall to the mouth of the fistula. Control angiography is then performed from both the left external carotid and common carotid artery, with no further arteriovenous shunting noted.

### 8:09 Disease Background.

Tentorial dural arteriovenous fistulas are overall rare entities accounting for about 4% of all intracranial dural fistulas. They carry a high risk of hemorrhage due to direct cortical venous drainage and venous hypertension, with a poor natural history without treatment.^1–6^ Transvenous embolization can be effective, especially when arterial routes are difficult or risky due to torturous feeders or dangerous anastomoses.^7–8^ It is critical to have careful preoperative study of the 3D angiography to optimize placement of microcatheters and obtain adequate transvenous support in order to navigate through tortuous, and often stenotic, draining veins.^5,9,10^

### 8:53 Clinical and Imaging Outcome.

There was no further arteriovenous shunting on control angiography after transvenous embolization. She remained at neurological baseline postoperatively and was subsequently discharged home after a short rehabilitation stay and currently has a modified Rankin of 3. She recently had her 6-month control angiography performed, demonstrating no further arteriovenous shunting from either the left external carotid or the left internal carotid artery injections, as shown here. Other vessels injected during complete 6-month control angiography at this time confirmed no arteriovenous shunting.

## Disclosures

The authors report no conflict of interest concerning the materials or methods used in this study or the findings specified in this publication.

## Author Contributions

Primary surgeon: Nguyen, Mattingly. Assistant surgeon: Barrera. Editing and drafting the video and abstract: Nguyen, Burns, Mendoza-Ayus, Kohli, Romiyo, Singh, Bender. Critically revising the work: Nguyen, Burns, Mendoza-Ayus, Barrera, Kohli, Singh, Bhalla, Bender, Mattingly. Reviewed submitted version of the work: Nguyen, Burns, Mendoza-Ayus, Barrera, Singh, Bender, Mattingly. Approved the final version of the work on behalf of all authors: Nguyen. Supervision: Nguyen, Barrera, Mattingly. Illustrator: Young.

## Supplemental Information

### Patient Informed Consent

The necessary patient informed consent was obtained in this study.
